# Predictor of Obese Mothers and Stunted Children in the Same Roof: A Population-Based Study in the Urban Poor Setting Indonesia

**DOI:** 10.3389/fnut.2021.710588

**Published:** 2021-12-06

**Authors:** Qonita Rachmah, Trias Mahmudiono, Su Peng Loh

**Affiliations:** ^1^Department of Nutrition, Faculty of Public Health, Universitas Airlangga, Surabaya, Indonesia; ^2^Department of Nutrition, Faculty of Medicine & Health Sciences, University Putra Malaysia, Selangor, Malaysia

**Keywords:** double burden malnutrition, obesity, stunting, socioeconomic disparity, nutrition

## Abstract

Stunting leads to the poor cognitive development, increases the risk of child mortality, and elevates the risk of non-communicable diseases. This study aimed to determine the magnitude of double burden of malnutrition (DBM) in the urban poor setting in Indonesia and investigate its predictors. This was a *cross-sectional* study involving 436 mothers proportionally chosen from 16 integrated health posts in Surabaya, Indonesia. The households were categorized into the two groups based on the body mass index (BMI) of mother and the height-for-age *z*-score (HAZ) of child; households without DBM and household with DBM. Energy, carbohydrate, protein, and fat intake were obtained using 24-h food recall and socioeconomic status was measured using a structured questionnaire. Data on socioeconomic status were educational level of mother and occupation, household income, and food expenditure. The prevalence of household with DBM was 27.5%; 12.4% pair stunted children and normal weight mother; 45.6% pair of overweight/obese mother and normal height children. The logistic regression analysis showed significant differences in the education level and occupation of mother, protein intake of the children, and fat intake of the mother between households with and without DBM. This study offers an important insight to improve the knowledge of mother related to the protein intake of children to reduce stunting risk and fat intake of mother to prevent over-nutrition.

## Introduction

Ending all forms of malnutrition, such as stunting was one of the Sustainable Development Goals (1). However, many developing countries are suffering from the double burden of malnutrition (DBM) defined as the coexistence of under-nutrition and over-nutrition (overweight/obesity) or diet-related non-communicable diseases (NCDs) due to significant nutrition shifts (2–4). The DBM could occur at the individual level, household level, and population level in all the life cycles (5–7). The DBM brings several health consequences. Child malnutrition could lead to the poor cognitive development, and lower immune system thus increasing the risk of infection and child mortality (8, 9). Another finding explained that children malnutrition led to an increased risk of NCDs such as type 2 diabetes mellitus, high blood pressure, and cardiovascular diseases, which is also the impact of over-nutrition (10). At least 60% of death cases in the Southeast Asia Region were caused by NCDs (11). A similar trend was reported in Indonesia in which NCDs account for 59.5% of the leading causes of death, one out of two due to cardiovascular disease (12).

The WHO in 2016 reported the amount of malnutrition across the globe, 155 million children were stunted and 1.9 billion adults were overweight (4). Simultaneously, Indonesia national survey has reported that the prevalence of overweight and obese in the adults was doubled from 19.1% in 2007 to 35.4% in 2018 with higher prevalence found in women than men, while the stunted children accounted for 30.8% (13). In the long term, stunted condition increases the risk of reducing the cognitive abilities, school performance, and potential income in the future (8, 14).

Within the peer-reviewed literature, several factors contribute to the occurrence of DBM at the household level. Popkin explained that the changes in dietary patterns and nutritional intake to modern lifestyle during economic and social development in the developing countries might contribute to the double burden malnutrition phenomenon (15). That nutrition shifts lead to several nutrition problems at the household level related to urbanization. Further, when urbanization occurs, most households increase their income level, and more food will be available in terms of quantity but not in quality (16). High density foods are high in energy but low in protein, and micronutrients could affect the growth of child and are linked to stunting (17). On the other hand, foods that are high in energy might also cause overweight/obesity among mothers under five children in the same household, especially coupled with low physical activity. A previous study in the Urban Surabaya revealed that 70.2% of mothers were overweight/obese, 36.4% of children were stunted, and 24.7% of households were DBM (18).

The results of the study in Guatemala showed that families in the third quintile of socio economic status (SES) had the highest prevalence of multiple nutritional problems 22.7% (19). This evidence indicates that at the household level, in the absence of food insecurity and economic shortages, behavioral factors such as food distribution and food diversity are allegedly associated with a high prevalence of the DBM. A few studies with mixed results, less sensitive measurement, and small sample size elaborate on the causes of the DBM in urban poor setting in Indonesia. Thus, our study aims to elaborate the causes of DBM in an urban poor environment in Indonesia with a larger sample size and more sensitive measurement, such as the use of Asia-pacific BMI cut-off points to determine overweight/obese.

## Method

### Study Design and Participants

This cross-sectional study was conducted on randomly selected 428 mothers who had under five children through proportionally random sampling from 16 integrated health posts (*Posyandu*) in Sidotopo district, Surabaya, Indonesia. The power for this study was 80% with a 95% *CI* or 5% alpha level and the population estimate of children under 5 years old in the Surabaya City used 2018 data (*n* = 213,590 children) (20). Assuming 50% response distribution, the minimal sample needed was 384 participants. Accounting for a 10% non-response rate, we surveyed 436 households with mothers and at least one child between 2 and 5 years of age. Excluding cases with missing data or extreme values (21), 428 households were analyzed. Inclusion criteria considered apparently healthy mothers with no known illness that required specific diet and having children aged 0–60 months old. The participants with known chronic illness (diabetes, cardiovascular diseases, kidney diseases, and liver diseases) and physical disabilities were excluded from this study. Children with known genetic disorders were also excluded.

This study was conducted according to the guidelines laid down in the Declaration of Helsinki and all the procedures involving human subjects/patients were approved by the Independent Ethics committee of the Faculty of Public Health, Universitas Airlangga (IRB number: KEPK-512). The written informed consent was obtained from all the subjects. The mothers were informed about their right to withdraw from this study anytime.

### Study Dimension

The socioeconomic profile of mothers and under-five children, such as date of birth, age, education background of mother and father, number of children, type of family, occupation of mother, and family income were collected using a structured questionnaire. Macronutrient intake of both mother and child was assessed by a trained nutritionist using 1 day 24-h food recall.

### Questionnaire Administration

The purpose of the questionnaire administration was explained to the mothers once their permission was obtained. They were informed about the participation in this study was voluntary and they were free to withdraw at any stage of this study. The interview was done by a trained nutritionist and was done at mother's home taking about 40–60 min.

### Anthropometric Measurements

The participant's body weight was measured in light clothing using OMRON digital weighing scale Model 375-HBF (OMRON Corporation, Japan) to the nearest 0.1 kg by adjusting the average clothes weight of 0.5 kg. A portable SECA stadiometer model 213 (SECA, Germany) was used to measure the height to the nearest 0.1 cm. Body mass index (BMI) was calculated and then categorized mothers based on their nutritional status. We use BMI category for the Asia-Pacific population as follow: normal (18.5–23.49 kg/m^2^), overweight (23.5–24.99 kg/m^2^), and obese (>25 kg/m^2^) (22).

For children, the anthropometric measurements include weight and length/height. The weight and height of child were measured using the same device as used for mothers, but length measurement for those ages <2 years was done using SECA length-board. Length/height *z*-scores for age and sex to define stunting were calculated using WHO Anthro software version 1.03. The body height status was classified using the WHO growth reference charts for age 0–5 years. The data were entered and analyzed using the SPSS version 23. The data were classified according to length/height *z*-scores for age and sex as normal (>-2 SD), stunted (−1 to −2 SD), and very stunted (<-3 SD) (23). The anthropometric measurements were collected after the administration of the questionnaire to avoid bias in responses while answering the questionnaire.

A household with DBM was defined as coexistence between overweight/obese mother based on BMI and stunted children based on the height-for-age *z*-score (HAZ) in one household (4).

### Dietary Analysis

Dietary data were collected using 24-h food recall in weekdays (Monday to Friday) by using the interview by trained nutritionist. Daily intake of respondents was then analyzed using *Nutrisurvey* software completed with the Indonesian Food Database. To ensure its accuracy, data were also checked for its under- or over-reporting before being statistically analyzed.

### Statistical Analysis

Descriptive analysis of socioeconomic and macronutrient intake of the mother and children under-5 years was done at first. The chi-square and ANOVA tests were used to look up the difference of socioeconomic characteristics and macronutrient intake. Then, logistic regression analysis was performed to compare the socioeconomic status and macronutrient intake between households with and without the DBM with 95% *CI*.

## Results

In this study, 436 participants were employed. However, eight respondents could not complete the anthropometric measurements of their children, so they were dropped out of the analysis. Having 428 participants of completed pair of mother and children in the final analysis, the study drop-out rate was accounted for 1.9%.

The observations for socioeconomic profile are presented in Table 1. The ANOVA test revealed that compared with the nutritional status of mother, there was a significant difference in terms of age of mother (*p* < 0.001), number of children in the household (*p* < 0.001), and daily fat intake of the mother (*p* = 0.016), but no difference in the terms of age of children (*p* = 0.392), and daily intake of energy, carbohydrate, and protein (*p* = 0.100*, p* = 0.235, *p* = 0.137, respectively). Most obese mothers were having older age, low educational status, higher number of children in the family, and higher daily fat intake (Table 1). Apart from fat intake, the intake of energy, protein, and carbohydrate did not show any significant difference.

**Table 1:** Socio-economic profile of household.

**Variables**	**Normal weight** **(BMI 18.5–23.49 kg/m^2^)**	**Overweight** **(BMI 23.5–24.99 kg/m^2^)**	**Obese** **(BMI >25 kg/m^2^)**	**Total** ***n* (column %)**	***P*** **value**
	***n*** **(row %)**	***n*** **(row %)**	***n*** **(row %)**		
Total	109 (25.5)	67 (15.7)	252 (58.9)	428 (100.0)	
Mother's age (years) (mean ± SD)	28.4 (6.0)	30.9 (6.2)	31.1 (7.1)	30.9 (6.9)	0.000^*^
Children's age (months) (mean ± SD)	29.4 (16.1)	30.7 (15.5)	31.9 (15.7)	31.1 (15.5)	0.392
Maternal education					
Low (no schooling or elementary school)	28 (17.2)	25 (15.3)	110 (67.5)	163 (38.1)	0.032^*^
Middle (junior high school)	32 (29.6)	18 (16.7)	58 (53.7)	108 (25.2)	
High (senior high school or college/university)	49 (31.2)	24 (15.3)	84 (53.3)	157 (36.7)	
Paternal education					
Low (no schooling or elementary school)	29 (22.1)	16 (12.2)	86 (65.6)	131 (30.6)	0.253
Middle (junior high school)	73 (26.3)	47 (16.9)	158 (56.8)	278 (65.0)	
High (senior high school or college/university)	7 (36.9)	4 (21.1)	8 (42.1)	19 (4.4)	
Number of children (mean ± SD)	1.8 (0.88)	1.7 (1.0)	2.2 (1.2)	2.2 (1.1)	0.000^*^
Maternal occupation					
Working	25 (22.7)	21 (19.1)	64 (58.2)	110 (25.7)	0.457
Not working	84 (26.4)	46 (14.5)	188 (59.1)	318 (74.3)	
Household's monthly income					
Low (≤Indonesian Rupiah/IDR 15,00,000 or U$150)	31 (25.8)	19 (15.8)	70 (58.3)	120 (28.0)	0.870
Medium (>IDR 15,00,000–2,500,000 or >U$150–250)	33 (22.8)	25 (17.2)	87 (60.0)	145 (33.9)	
High (>IDR 25,00,000 or >$250)	45 (27.6)	23 (14.1)	95 (58.3)	163 (38.1)	
Daily intake of mother's					
Energy (kcal/day) (mean ± SD)	1195.8 (452.6)	1275.9 (544.1)	1140.2 (445.2)	1175.1 (464.5)	0.100
Carbohydrate (gr/day) (mean ± SD)	151.6 (72.3)	158.7 (66.3)	143.2 (65.0)	147.5 (67.4)	0.235
Protein (gr/day) (mean ± SD)	44.1 (18.9)	49.1 (24.7)	43.8 (20.1)	44.7 (20.6)	0.137
Fat (gr/day) (mean ± SD)	45.3 (22.4)	45.7 (28.0)	43.3 (23.3)	45.3 (24.1)	0.016^*^

* *Variables are significantly associated with mother's nutritional status at level α < 0.05 obtained with Chi-square and ANOVA test*.

Most of the households were nuclear families (50.6%) with mean age of the mother was 30.88 years, mean age of children was 31.2 months, mean number of children in the household was 2.16 with minimum number of children was 1 (32.9%) and the maximum was 8 (0.2%) (not directly shown in the table).

Significant different using the *chi* square analysis based on the nutritional status of mother was also found in terms of maternal education (*p* = 0.032), but not in paternal education (*p* = 0.253), maternal occupation (*p* = 0.457), and monthly income of household (*p* = 0.870). Specifically, most of the mothers graduated from high school (33.9%), not working (74.3%); while most of the working mothers worked as entrepreneurs (9.3%). Monthly income of the household was mostly categorized as high income (>IDR 2,500,000 or >$250) (38.1%) with mean monthly income was IDR 2,755,343 + 2,220,195. However, only 131 households answered their mean monthly income in numbers, another 297 did not answer their mean monthly income.

Table 2 shows the characteristics of children under-5 years based on the HAZ of the children. The findings illustrate the significant difference in terms of maternal education (*p* = 0.005), paternal education (*p* = 0.018), number of children in the household (*p* = 0.040), and household monthly income (*p* = 0.025). It is revealed that the low educational background of parents with more children in the household tends to lead to lower monthly income.

**Table 2:** The descriptive characteristics of children under-5 years.

**Variables**	**Children' height-age z score (HAZ)**				**P*value***
	**Normal height** **(2SD−2SD)**	**Stunted** **(HAZ −2SD–−3SD)**	**Very stunted** **(SD <-3SD)**	**Total** **n (column %)**	
	**n (row %)**	**n (row %)**	**n (row %)**		
Mother's age (years) (mean ± SD)	31.1 (6.9)	20.9 (6.6)	30.0 (6.9)	30.9 (6.9)	0.477
Children's age (months) (mean ± SD)	31.0 (16.3)	31.6 (14.5)	30.6 (15.7)	31.1 (15.5)	0.891
Maternal education					
Low (no schooling or elementary school)	84 (51.15)	46 (28.2)	33 (20.2)	163 (38.1)	0.005^*^
Middle (junior high school)	61 (56.5)	33 (30.6)	14 (13.0)	108 (25.2)	
High (senior high school or college/university)	109 (69.4)	24 (15.3)	24 (15.3)	157 (36.7)	
Paternal education					
Low (no schooling or elementary school)	63 (48.1)	38 (29.0)	30 (22.9)	131 (30.6)	0.018^*^
Middle (junior high school)	177 (63.7)	63 (22.7)	38 (13.7)	278 (65.0)	
High (senior high school or college/university)	14 (73.7)	2 (10.5)	3 (15.8)	19 (4.4)	
Number of children (mean ± SD)	2.05 (1.1)	2.4 (1.2)	2.3 (1.1)	2.2 (1.1)	0.040^*^
Maternal occupation					
Working	62 (56.4)	33 (30.0)	15 (13.6)	110 (25.7)	0.205
quad Not working	192 (60.4)	70 (22.0)	56 (17.6)	318 (74.3)	
Household's monthly income					
Low (≤Indonesian Rupiah (IDR) 1,500,000 or U$150)	61 (50.8)	36 (30.0)	23 (19.2)	120 (28.0)	0.025^*^
Medium (>IDR 1,500,000–2,500,000 or >U$150–250)	81 (55.9)	35 (24.1)	29 (20.0)	145 (33.9)	
High (>IDR 2,500,000 or >$250)	112 (68.7)	32 (19.6)	19 (11.7)	163 (38.1)	
Daily intake of children					
Energy (kcal/day) (mean ± SD)	993.1 (441.3)	1026.8 (465.7)	859.5 (429.9)	979.0 (447.8)	0.720
Carbohydrate (gr/day) (mean ± SD)	121.3 (61.1)	123.5 (62.3)	106.4 (61.5)	119.3 (61.6)	0.833
Protein (gr/day) (mean ± SD)	36.8 (20.1)	35.5 (19.3)	28.2 (16.8)	35.0 (19.6)	0.269
Fat (gr/day) (mean ± SD)	42.6 (30.8)	40.2 (21.3)	36.3 (20.2)	41.0 (27.3)	0.863

* *Variables are significantly associated with children's HAZ at level α < 0.05 obtained with Chi-square and ANOVA test*.

There was a coexistence of child undernutrition (stunted) with maternal overweight/obesity shown by this study. As much as 28.0% households experienced the DBM (stunted children and overweight/obese mothers), 46.5% pair of stunted children with normal weight mothers, and 12.6% pair of normal height children and overweight/obese mothers as shown in Table 3.

**Table 3:** Coexistence of under- and over-nutrition in the same household, Surabaya, Indonesia.

	**Frequency**	
	**N**	**%**
Normal pair	55	12.9
Stunted children + normal weight mother	199	46.5
Overweight/obese mother + normal height children	54	12.6
Stunted children + overweight/obese mother	120	28.0
Total	428	100

Table 4 presents several variables that were significantly associated with the DBM measured as stunted children and overweight/obese mothers when tested using logistic regression. Compared with household without DBM, households with DBM were significantly having lower maternal education (*p* = 0.000; *OR* = 3.174; 95% *CI* = 1.876–5.370), lower paternal education (*p* = 0.001; *OR* = 2.517; 95% *CI* = 1.380–3.372), higher number of children in the same household (*p* = 0.000; *OR* = 1.116; 95% *CI* = 1.024–1.208), and lower household's monthly income (*p* = 0.009; *OR* = 2.043; 95% *CI* = 1.200−3.479). In addition, DBM also more prevalent in household where mothers are working compared to non-working mothers (*p* = 0.042; *OR* = 0.632; 95% *CI* = 0.380–1.051). Children in DBM household were also significantly having a lower intake of protein compared to the household without DBM. To be specific, children with low protein intake had 1.364 higher risks to contribute to the occurrence of the DBM (*OR* = 1.364; 95% *CI* = 1.276–1.451). However, there was no significant difference observed between energy, carbohydrate, and fat intake between households with and without DBM. On the other hand, the mother's intake of fat was higher in the household with DBM than the household without DBM (*p* = 0.000; *CI* = 1.314; 95% *CI* = 1.222–1.405).

**Table 4:** Comparison of socio-economic and macronutrient intake between household with and without the double burden of malnutrition (DBM).

**Variables**	**Without DBM**	**DBM**	**Total**	***P*** **value**	**Odds Ratio (95% CI)**
	***n*** **(row %)**	***n*** **(row %)**	***n*** **(column %)**		
Mother's age (years) (mean ± SD)	30.6 (6.9)	31.5 (6.7)	30.9 (6.9)	0.084	0.813 (0.620–1.067)
Children's age (months) (mean ± SD)	30.8 (16.3)	31.8 (14.4)	31.1 (15.5)	0.447	1.001 (0.988–1.015)
Maternal education					
Low (no schooling or elementary school)	100 (61.3)	63 (38.7)	163 (38.1)	Reference	
Medium (junior high school)	77 (71.3)	31 (28.7)	108 (25.2)	0.093	1.565 (0.928–2.639)
High (senior high school or college/university)	131 (83.4)	26 (16.6)	157 (36.7)	0.000	3.174 (1.876–5.370)
Paternal education					
Low (no schooling or elementary school)	79 (60.3)	52 (39.7)	131 (30.6)	Reference	
Medium (junior high school)	213 (76.6)	65 (23.4)	278 (65.0)	0.001^*^	2.517 (1.380–3.372)
High (senior high school or college/university)	16 (84.2)	3 (15.8)	19 (4.4)	0.055	3.511 (0.974–12.649)
Number of children (mean ± SD)	2.0 (1.1)	2.5 (1.2)	2.2 (1.1)	0.000^*^	1.116 (1.024–1.208)
Maternal occupation					
Working	74 (67.3)	36 (32.7)	110 (25.7)	Reference	
Not working	234 (73.6)	84 (26.4)	318 (74.3)	0.042^*^	0.632 (0.380–0.899)
Household's monthly income					
Low (≤Indonesian Rupiah (IDR) 15,00,000 or U$150)	78 (65.0)	42 (35.0)	120 (28.0)	Reference	
Medium (>IDR 15,00,000–25,00,000 or >U$150–250)	101 (69.7)	44 (30.3)	145 (33.9)	0.421	1.236 (0.738–2.070)
High (>IDR 25,00,000 or >$250)	129 (41.9)	34 (20.9)	163 (38.1)	0.009^*^	2.043 (1.200–3.479)
Daily intake of mother's					
Energy (kcal/day) (mean ± SD)	1187.8 (456.2)	1143.2 (487.6)	1175.1 (464.5)	0.786	1.000 (0.998–1.002)
Carbohydrate (gr/day) (mean ± SD)	148.8 (67.6)	144.9 (66.9)	147.5 (67.4)	0.760	0.999 (0.989–1.010)
Protein (gr/day) (mean ± SD)	45.4 (19.6)	42.7 (23.1)	44.7 (20.6)	0.557	0.994 (0.974–1.013)
Fat (gr/day) (mean ± SD)	43.6 (24.7)	45.8 (25.8)	45.3 (24.1)	0.000 ^*^	1.314 (1.222–1.405)
Daily intake of children					
Energy (kcal/day) (mean ± SD)	992.9 (449.5)	938.8 (442.4)	979.0 (447.8)	0.116	1.003 (1.000 – 1.006)
Carbohydrate (gr/day) (mean ± SD)	121.2 (61.9)	114.0 (60.9)	119.3 (61.6)	0.380	0.989 (0.977–1.020)
Protein (gr/day) (mean ± SD)	36.3 (19.8)	31.5 (18.5)	35.0 (19.6)	0.000^*^	1.364 (1.276–1.451)
Fat (gr/day) (mean ± SD)	42.2 (29.2)	37.8 (21.4)	41.0 (27.3)	0.262	0.977 (0.947–1.007)

* *The variables are significantly associated with DBM status, the p-values obtained with logistic regression and binary logistic*.

## Discussion

Although the prevalence of stunted children and adult women overweight/obesity have been established in Indonesia, the coexistence of stunted children and overweight/obese mothers within the same household has not been examined, specifically in the poor urban area. Therefore, this study makes a significant contribution to the body of knowledge in this area. Our objective was to analyze the predictors of the DBM among the urban poor households. The predictors of DBM that we analyzed were socioeconomic status, such as the age of mother and child, maternal and paternal education, number of children in the household, maternal occupation, and household monthly income and macronutrient intake including energy, carbohydrate, protein, and fat intake of mother and child. DBM was defined as pair of overweight/obese mother and stunted child. These results in parallel with the hypotheses stated by the WHO that socioeconomic disadvantage and poverty and lifestyle affect the nutritional intake (4).

We found the prevalence of stunted children was 40.7% and overweight/obese mother was 74.5%. Those numbers were higher than the national prevalence of stunted and adult female overweight/obese (30.8% vs. 21.6%, respectively) (18). Moreover, our result revealed the household DBM as much as 27.5% higher than a study conducted in another urban setting in Indonesia (24.7%). However, previous study in Indonesia defined overweight/obese mothers using BMI cut-off of 25 kg/m^2^, while our study use BMI cut-off for Asian population which categorize overweight for those with BMI >23.5 kg/m^2^ (22). Using lower cut off to detect overweight/obese could be more beneficial for screening a high-risk individual, more sensitive in case finding, and being able to use for early treatment. Also, WHO stated that BMI and comorbidities association are differ between populations, thus using population-specific cut off is considerably better.

Factors related to obesity among mothers in our study were age, educational background, number of children, and fat intake. Brown explained that overweight and obesity are a complex and chronic condition resulting from interaction between individual, physiological, and environmental that affects the type, frequency, and quantity of food and beverages consumed, and the metabolic change of body (24). Several risk factors interact with one another, resulting in the “globesity” epidemic. In simple terms, obesity arises due to the imbalance energy; meaning calories intake are exceeded calories expended. Higher calories intake has been correlated with high-income environment; but nowadays, this trend is also shown in low- and middle-income household, as seen in this present study. So, stated earlier that obesity is due to complex interaction between several risk factors, apparently personal behaviors in response to those risk factors play a crucial role in obesity (25).

The educational background seems to correlate with the higher fat intake whereas age correlates with the number of children. Compared to other macronutrients, fat is known to have the highest calorie per gram of fat (9 kcal/g), thus consuming a higher amount of fat could escalate caloric intake. Interestingly, we found that mothers often consume the leftover of their children as some believe that food should not be wasted or called “*mubadzir*.” The complementary food composition of a child has higher fat content (around 50%), so does it leftover, which based on our view, might contribute to higher fat intake among the mothers. Moreover, a higher number of children also contribute to the high prevalence of obesity among mothers. A National Health Survey among 1,354 Iranian women also revealed a similar result with a 16% increase in the odds of obesity in women with an additional number of children after adjusted with potential confounding (26). Another cohort study among 2,035 women in Utah revealed 11% higher risk of obesity with each additional live birth independent of socioeconomic status (27). Several studies have explained that three mechanisms-mediated pregnancy-related weight gain; first, physiological changes such as hormonal alterations secondary to fewer ovulatory cycle (28), increased glucocorticosteroid activity (29), and insulin resistance associated with pregnancy (30) and the second is due to weight retention after pregnancy and the last one is due to lower physical activity after pregnancy (31).

Our study also found a high number of stunted children, 46.5%. This prevalence exceeded a national prevalence (13). However, we noted that this prevalence was found in the slum area of Surabaya with the highest prevalence of stunting in the city; hence, it was overestimated to the prevalence of child stunting in Surabaya as a whole. The factors related to stunted under-five children were maternal and paternal education, the number of children, and household income. An analysis of 62 national Demographic Health Survey observed the same result. In contrast, higher stunting prevalence was found in both lower maternal and paternal education after adjusting for child sex, age, country-level fixed effects, childbirth order, wealth quintile, maternal age, and local area characteristics (32). A potential explanation of maternal and paternal education increases the risk of stunting is mediated by the income; in which higher education level enables parents to earn higher incomes subsequently improved child health (33). This hypothesis was proven in our study as we did further analysis and revealed that higher income correlated with higher education level on both mother and father. Further, household income is also related to stunted, which implies the mutual relationship between parents' education and income and stunting. This result is consistent with a community-based study in Indonesia and Bangladesh (34, 35), also case-control study in the South Ethiopia which demonstrated a household with a high number of under-five children were four times more likely to develop stunting compared to those living with least number of under-five children (36). The higher number of children increased a higher competition for available food compared to the smaller family members, affecting dietary diversity among individuals and could lead to stunting (36, 37).

An overweight/obese mother and stunted child is a form of a DBM in a household which could bring much more burden in a household, both health and economic wise. This study obtains a pair of overweight/obese mother and stunted child as much as 28%, higher than other countries such as Ecuador 13.1% (38), Egypt 16.0% (39), and Guatemala 16.8% (40). However, a note should be taken since those studies using a BMI cut-off point of 25.0 kg/m^2^ to define overweight, while our study using 23.5 kg/m^2^ as a cut-off point thus, we found a higher DBM number. The factors that significantly predict DBM were maternal education, paternal education, number of children, maternal occupation, household monthly income, fat intake of mothers, protein intake of children. We believed that those predicting factors of household DBM interact with one another resulting in a different nutritional status among people in the same household. As previously mentioned, maternal and also paternal education might affect the family income ultimately leads to improve the child's intake (32, 34, 35). Our result also confirmed a study in the Latin America that found that education level was a protective factor to DBM (41). Furthermore, a review suggested that low maternal education was linked to poor feeding practice, thus increasing the risk of child stunting. In other words, education proved as the protective factor to DBM (42). Moreover, it is also strengthened by the findings that mothers who are not working increase 6% likelihood of household DBM characterized by overweight/obese mothers and stunted children. A possible explanation is that mothers who are not working might spend more sedentary activity than those working and also be related to family income. Mothers who are not working tend to be in low-income families and are not able to fulfill their child's nutritional intake (43).

Household monthly income also proved to increase the likelihood of DBM by 5%. The minimum wage for workers that should be obtained according to the location of this study is IDR 4.2 million or USD 266 (44) but we found that 61.9% household was still below the minimum wage. Low income directly affects the household food security and might contribute to the nutritional status of the family. However, we did not measure food security in this study. Interestingly, our study also successfully captures that mother's fat intake and child's protein intake were correlated with household DBM. Nutrient intake of family members depends on the food available at home. In Asian setting, mothers mostly have a central role as food providers; with limited education related to healthy eating behavior, mothers could provide unbalanced food for the family and impact nutritional status (45). As an example of our study, children in DBM household have 5 g less protein intake compared to children in non-DBM household (32 vs. 36 g, respectively). This result is supported by a study presenting a high rate of stunting among regions with a high prevalence of protein inadequacy (35.4 g in South Asia and 34.7 in Sub-Saharan Africa) (46). Protein intake is crucial for the children's linear growth and prevents stunting. First, adequate-protein intake promotes a hormonal growth called insulin-like growth factor-1, and then induces a normal linear growth (47). Furthermore, a study among rural Malawi children with 62% stunting prevalence explains that insufficient intake of essential amino acids represses protein and lipid synthesis through mammalian target of rapamycin complex 1 (mTORC1) and general control nonderepressible 2 (GCN2) growth regulatory pathways resulting in the cell growth restriction and further cause stunting (48).

One of the strengths of our study was a wide range of predictors analyzed causing the DBM in the urban poor setting area. Wide range predictors could give a better understanding of what causes DBM thus can be used to develop a strategic intervention. However, this cross-sectional study did not imply causation between variables which become a limitation. Moreover, the use of one-day 24-h food recall also became a limitation of this study. To overcome this limitation, food recall was done by trained nutritionist and recall was done face-to-face. Further study can also widen the definition of DBM using the malnutrition cases, not only stunting case.

## Data Availability Statement

The original contributions presented in the study are included in the article/supplementary material, further inquiries can be directed to the corresponding author.

## Ethics Statement

The studies involving human participants were reviewed and approved by the Independent Ethics Committee of Faculty of Public Health, Universitas Airlangga. The participants provided their written informed consent to participate in this study.

## Author Contributions

All authors listed have made a substantial, direct, and intellectual contribution to the work and approved it for publication.

## Funding

This study was funded by the Universitas Airlangga fiscal year 2018 under the *Hibah Mandat* research scheme.

## Conflict of Interest

The authors declare that the research was conducted in the absence of any commercial or financial relationships that could be construed as a potential conflict of interest.

## Publisher's Note

All claims expressed in this article are solely those of the authors and do not necessarily represent those of their affiliated organizations, or those of the publisher, the editors and the reviewers. Any product that may be evaluated in this article, or claim that may be made by its manufacturer, is not guaranteed or endorsed by the publisher.
